# Supplementary material for: Selective Control by Pistacia vera L. and Its Carotenoid Zeaxanthin on SARS-CoV-2 Virus
Source: Int J Mol Sci. 2025 Feb 15;26(4):1667. doi: 10.3390/ijms26041667 (PMC11855127; doi:10.3390/ijms26041667)
Supplement: Supplementary file 1 [file ijms-26-01667-s001.zip › ijms-3442842-supplementary.pdf]

International Journal of
Molecular Sciences

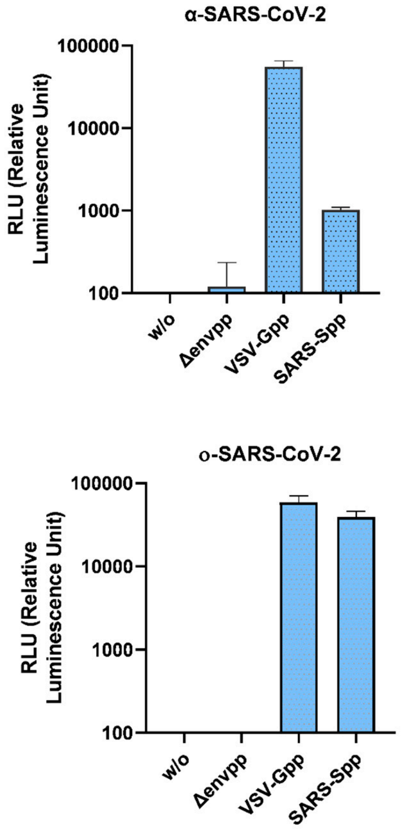

Figure S1. Quantification of the infectivity of viral pseudotyped particles. The luciferase activity was measured in Vero cells 72 h post-infection with no envelope pseudotyped particles (Δenvpp), VSV-G-pseudotyped particles (VSV-Gpp), and α and o-SARS-CoV-2 S-pseudotyped particles (SARS-Spp). Experiments were performed in triplicates, and data represent the average RLU of three independent experiments (n = 3).

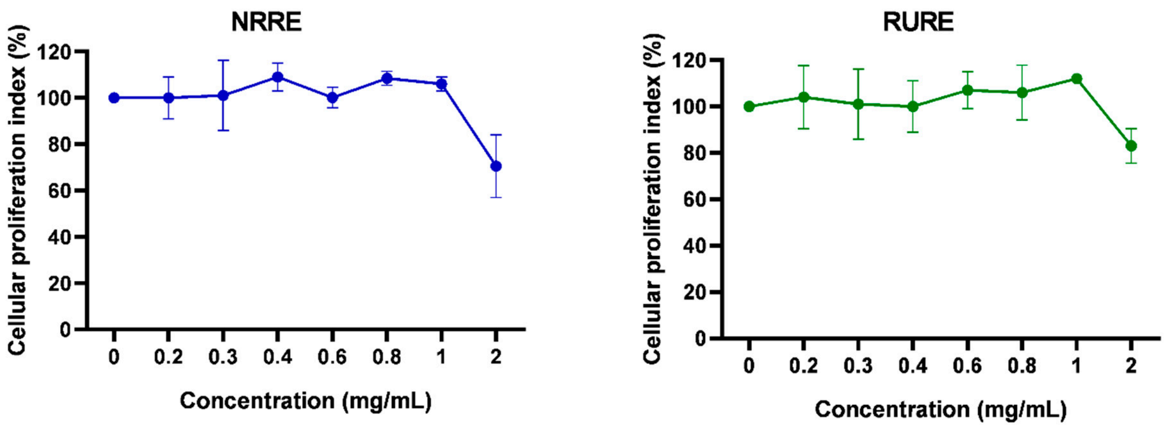

Figure S2. Cytotoxicity of A549 following treatment with NRRE and RURE. A549 cells were incubated with different concentrations of NRRE and RURE for 72 h. The absorbance was measured at 460 nm, and the % of cellular proliferation was calculated concerning the untreated cells. Data are expressed as a mean (SD) of at least three experiments.

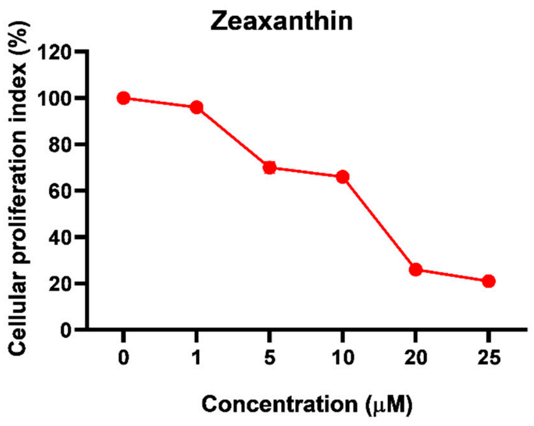

Figure S3. Cytotoxicity of A549 following treatment with zeaxanthin. A549 cells were incubated with different concentrations of zeaxanthin for 72 h. The absorbance was measured at 460 nm, and the % of cellular proliferation was calculated concerning the untreated cells. Data are expressed as a mean (SD) of at least three experiments.

Table S1. Half maximal cytotoxic concentration (CC_{50}) values of studied compounds on A549 cells.

Compounds	CC_{50}[a]
NRRE (mg/mL)	2.08
RURE (mg/mL)	2.12
ZEAXANTHIN (μM)	12.89

[a] CC_{50}: half maximal cytotoxic concentration

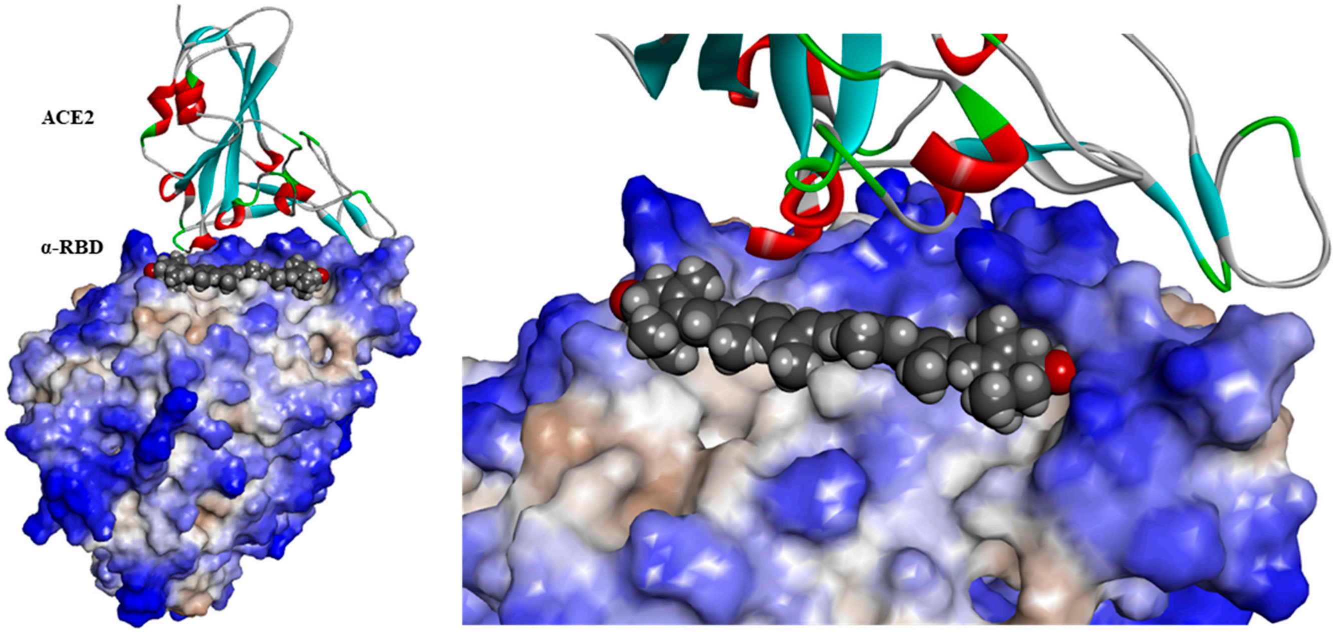

Figure S4. Hydrophobic surface α−RBD domain and human ACE2 in complex with zeaxanthin (gray sphere model) and close-up view.

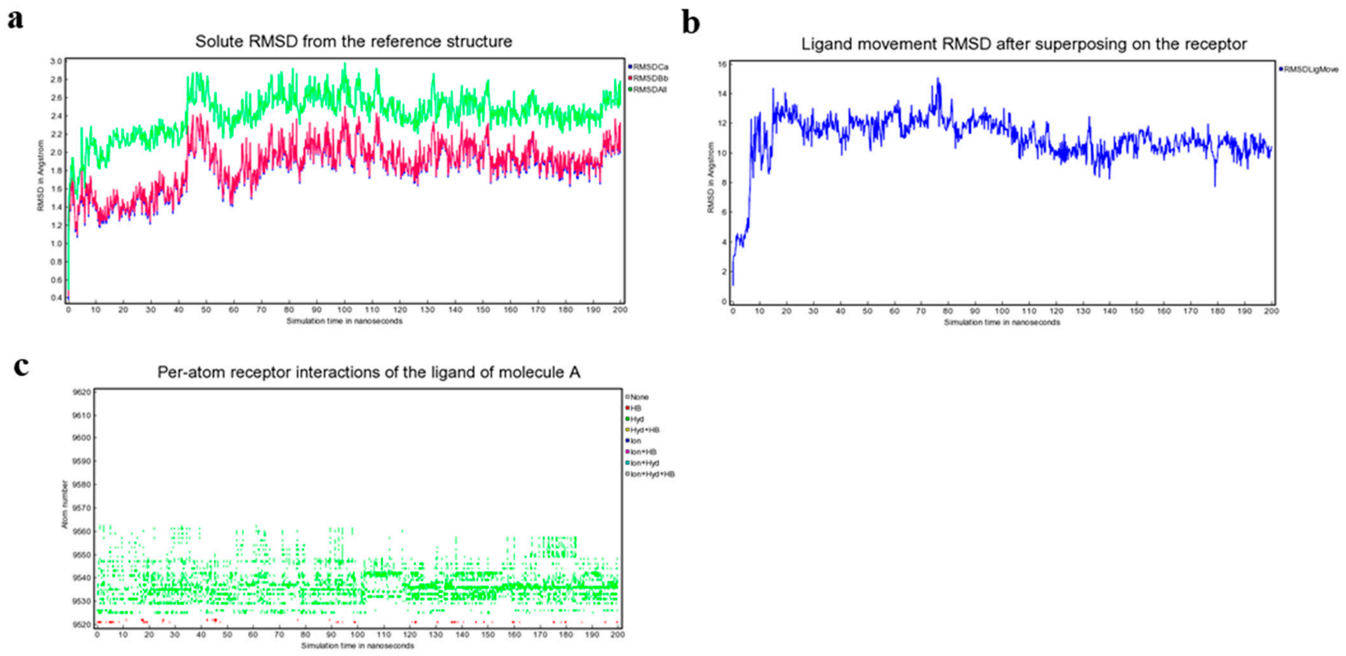

Figure S5. RMSDs of protein (a), RMSDs ligand (b), and per-atom receptor interactions of the ligand (c) of the zeaxanthin/α−RBD complex.

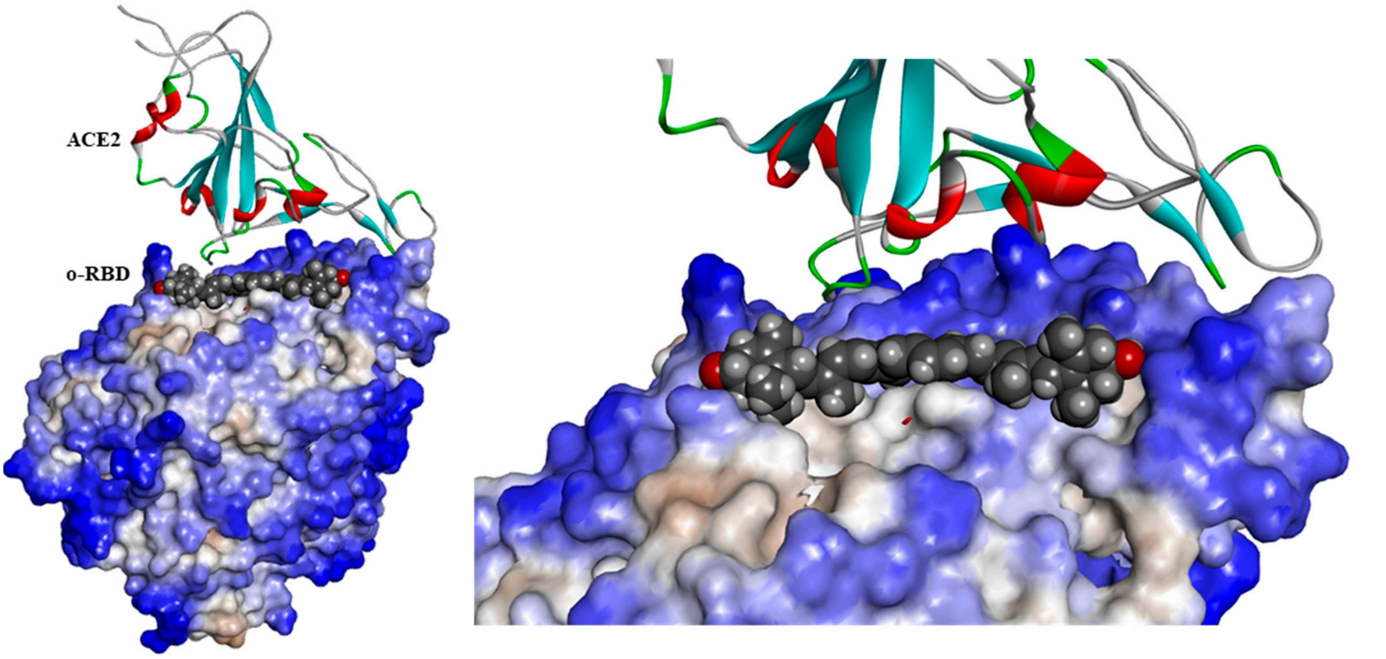

Figure S6. Hydrophobic surface o-RBD domain and human ACE2 in complex with zeaxanthin (gray sphere model) and close-up view.

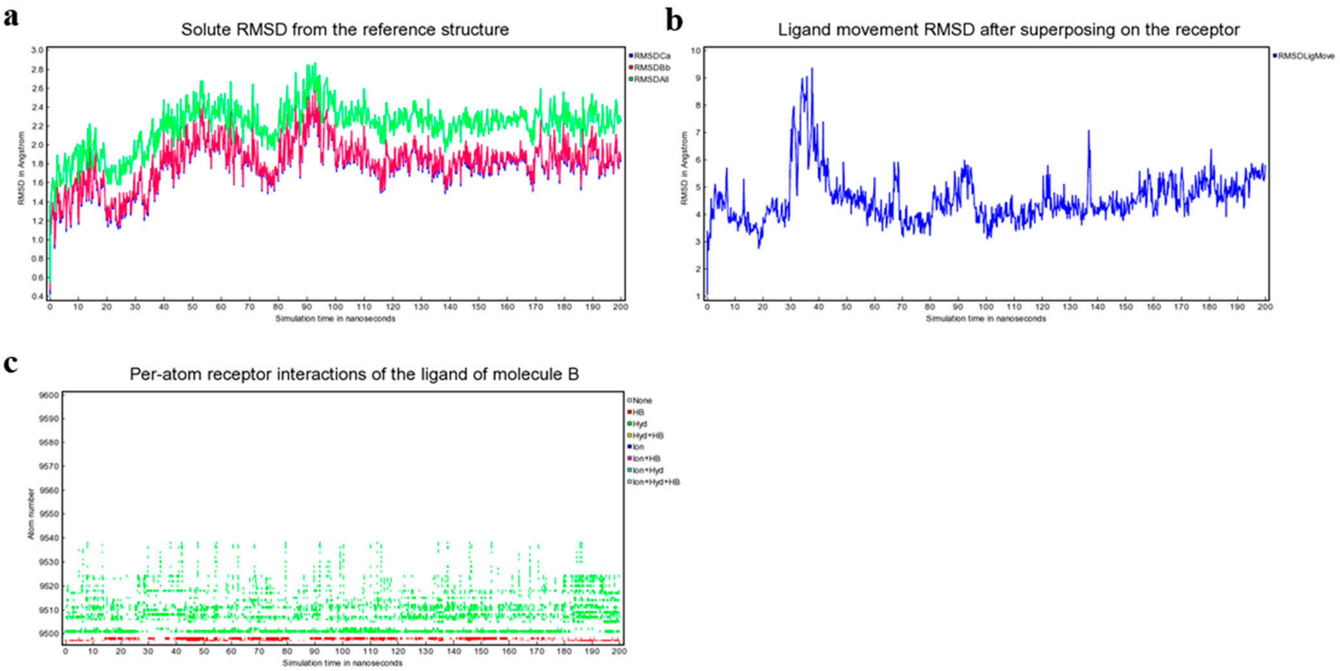

Figure S7. RMSDs of protein (a), RMSDs ligand (b), and per-atom receptor interactions of the ligand (c) of the zeaxanthin/o-RBD complex.

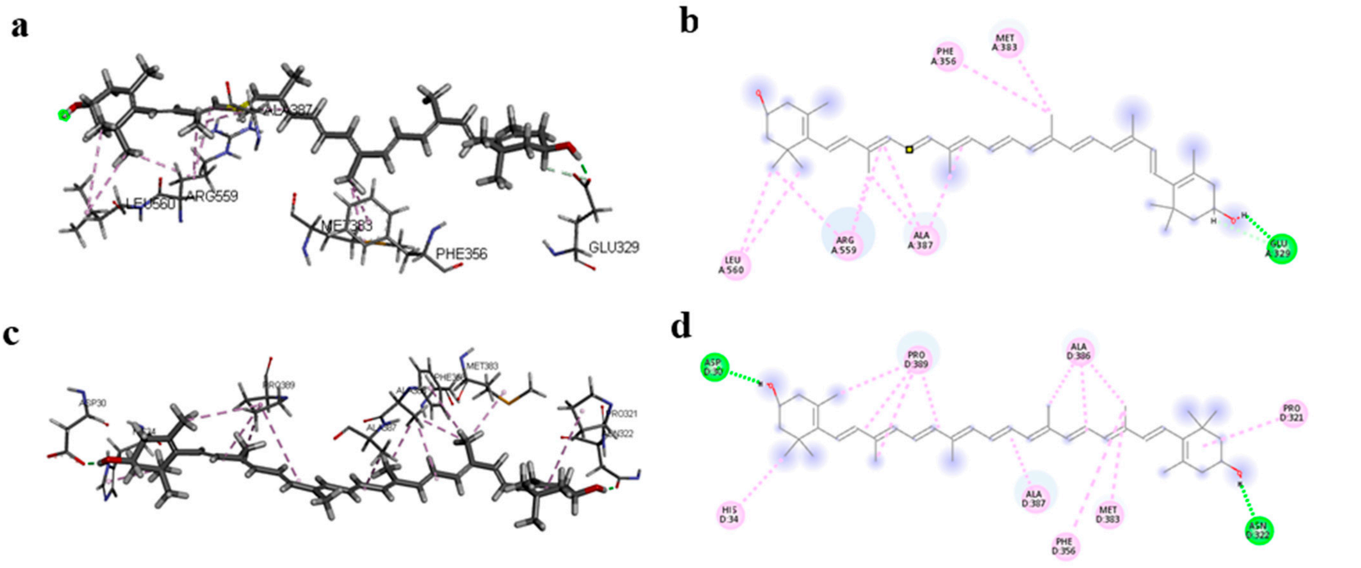

Figure S8. Interaction profile of the docked poses and 2D diagram interaction profile of zeaxanthin of the α-RBD (a, b) and o-RBD domain (c, d) after 200 ns of MD simulation.

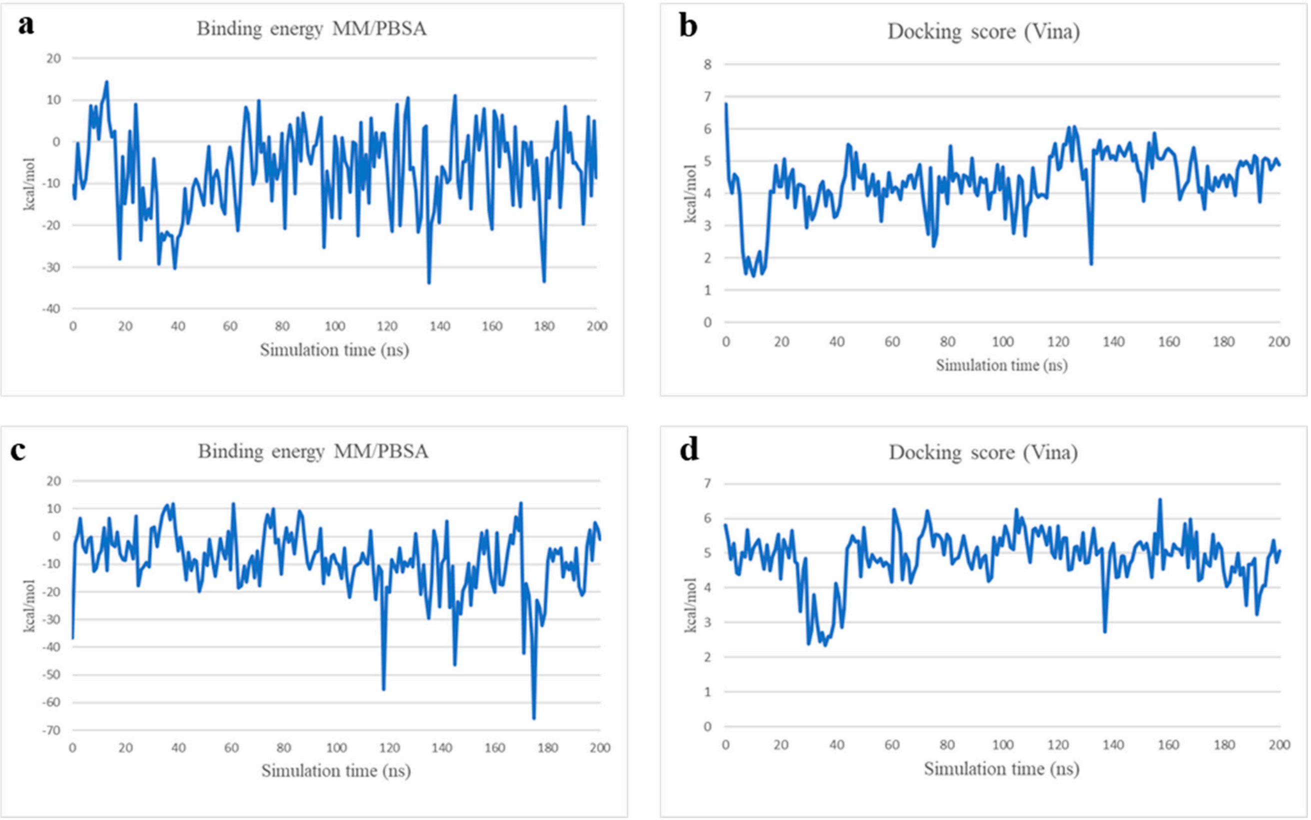

Figure S9. Binding free energy calculations of protein-ligand complexes obtained from MM-PBSA analysis and docking score calculation for α-RBD (a, b) and o-RBD (c, d).
